# Validation and application of the Non-Verbal Behavior Analyzer: An automated tool to assess non-verbal emotional expressions in psychotherapy

**DOI:** 10.3389/fpsyt.2022.1026015

**Published:** 2022-10-28

**Authors:** Patrick Terhürne, Brian Schwartz, Tobias Baur, Dominik Schiller, Steffen T. Eberhardt, Elisabeth André, Wolfgang Lutz

**Affiliations:** ^1^Clinical Psychology and Psychotherapy, University of Trier, Trier, Germany; ^2^Chair for Human Centered Artificial Intelligence, Augsburg University, Augsburg, Germany

**Keywords:** emotion recognition, process-outcome, video analyses, prediction, facial coding, software validation, software application

## Abstract

**Background:**

Emotions play a key role in psychotherapy. However, a problem with examining emotional states via self-report questionnaires is that the assessment usually takes place after the actual emotion has been experienced which might lead to biases and continuous human ratings are time and cost intensive. Using the AI-based software package Non-Verbal Behavior Analyzer (NOVA), video-based emotion recognition of arousal and valence can be applied in naturalistic psychotherapeutic settings. In this study, four emotion recognition models (ERM) each based on specific feature sets (facial: OpenFace, OpenFace-Aureg; body: OpenPose-Activation, OpenPose-Energy) were developed and compared in their ability to predict arousal and valence scores correlated to PANAS emotion scores and processes of change (interpersonal experience, coping experience, affective experience) as well as symptoms (depression and anxiety in HSCL-11).

**Materials and methods:**

A total of 183 patient therapy videos were divided into a training sample (55 patients), a test sample (50 patients), and a holdout sample (78 patients). The best ERM was selected for further analyses. Then, ERM based arousal and valence scores were correlated with patient and therapist estimates of emotions and processes of change. Furthermore, using regression models arousal and valence were examined as predictors of symptom severity in depression and anxiety.

**Results:**

The ERM based on OpenFace produced the best agreement to the human coder rating. Arousal and valence correlated significantly with therapists’ ratings of sadness, shame, anxiety, and relaxation, but not with the patient ratings of their own emotions. Furthermore, a significant negative correlation indicates that negative valence was associated with higher affective experience. Negative valence was found to significantly predict higher anxiety but not depression scores.

**Conclusion:**

This study shows that emotion recognition with NOVA can be used to generate ERMs associated with patient emotions, affective experiences and symptoms. Nevertheless, limitations were obvious. It seems necessary to improve the ERMs using larger databases of sessions and the validity of ERMs needs to be further investigated in different samples and different applications. Furthermore, future research should take ERMs to identify emotional synchrony between patient and therapists into account.

## Introduction

Emotions are a central component of human communication, form the basis of interpersonal relationships, indicate how we feel, provide feedback about internal states, and prepare impulses for action (1). Most mental disorders are characterized by some form of emotional impairment. Dysfunctional behaviors often result from difficulties in dealing with unpleasant feelings (2). Especially emotional disorders (e.g., depression) are characterized by frequent and intense negative emotions, a diminished sense of control and negative appraisal of specific emotions, as well as efforts to avoid emotions (3). Beyond that, patients with mental disorders seem to have problems expressing their emotions adequately. For instance, their facial activity is reduced and their ability to imitate emotional expressions is impaired, which makes it harder for them to establish healthy relationships (4–7). Therefore, focusing on emotions is transdiagnostically relevant to the therapeutic process and outcome over nearly all psychotherapeutic modalities (8).

In a meta-analysis including 42 studies, an averaged weighted effect size of *r* = 0.40 between patients’ emotional expressions and treatment outcome was found (9) indicating that stronger expressions of affect were associated with better outcomes. However, the authors point out that direct evidence of causality cannot be demonstrated with this correlational approach. Further findings suggest that emotions contain information about a patient’s underlying needs and motives (10) and that emotional empathy fosters the therapeutic relationship, which is associated with better treatment outcomes (11). Additionally, the affective experience during therapy which is a predictor of symptom reduction (12) has been shown to be characterized by negative valence and high arousal (13).

Since emotions are a central element of human interaction, emotion research has a long tradition. Wundt (14) was one of the first to distinguish between the two different aspects of valence (ranging from feeling pleasant to unpleasant) and arousal (ranging from feeling quiet to active). Many other definitions of significant emotion features followed, which differ primarily in the number of dimensions and their labeling (15). The circumplex model of emotions (16) is one of the most established theoretical models and describes emotions as a specific combination of the dimensions arousal and valence. These two dimensions are arranged orthogonally, resulting in a coordinate system in which emotions can be mapped. This model integrates non-verbal information multimodally, allowing easy determination of emotional expressions (17).

Besides these theoretical considerations of emotion classifications, there is a great variety of methods to assess emotions (e.g., self-reports, physiological measures, external observations of salient emotional cues). Regarding patients’ emotions–and their change over the course of therapy–research has so far been based mainly on subjective self-reports. Here, evidence for the association between patient emotions and treatment success was found (18–20). However, a problem with examining emotional states via self-report questionnaires is that the assessment usually takes place after the actual emotion has been experienced which might lead to biases. For a detailed review of possible reasons for faulty memories of emotional experiences, see Levine et al. (21). Their findings suggest that diagnostic and experimental tests based on self-reports of past emotions, and testimony concerning the emotional impact of past events, should be interpreted with caution, particularly when an individual’s report follows major changes in his or her goals and beliefs. Moreover, during the therapy session, emotions are usually expressed non-verbally and determined based on salient non-verbal cues. In particular, facial expression has proven to be a helpful indicator for emotion recognition (22). Manual external observational ratings of emotional expressions yielded results superior to self-report ratings [for an overview see (15)], however, they have to be considered a time-consuming procedure (23).

In this context, modern automated external observational methods such as artificial intelligence (AI) video analysis software seem a promising way to examine emotions. They are less time-consuming and less expensive than manual ratings, more objective than patients’ self-report ratings, and they can provide continuous ratings with a high number of measurement time points for the entire length of a psychotherapy session. Due to the high temporal resolution, the application of AI video analysis software supplies new opportunities to examine emotions in the context of psychotherapy. Moreover, AI video analysis software allows the analysis of psychotherapy processes without having to implement additional equipment (e.g., electrodes) in the psychotherapeutic setting. This makes it possible to recognize emotions in a naturalistic setting without influencing the therapeutic process. Furthermore, with the help of video analysis tools, it is possible to examine expressions of emotion without the previously mentioned possible distortions in human ratings. Accordingly, once a suitable emotion recognition model (ERM) for software-based examination has been trained, it can be applied to an unlimited number of later recorded therapy sessions. Candra et al. (24), for example, proposed to use automated emotion recognition tools give feedback to psychotherapists and thus enable them to pay more attention on contemplation of emotions in the reflection of the sessions. Available software varies with regard to the quality of emotion recognition (25, 26). However, in a review of deep-learning approaches, recognition accuracy averaged 72% (27). Real-life applications of software solutions for an automated and continuous emotion recognition still remain an open challenge as most software’s reliability is limited in naturalistic settings (28). There are only a few first studies which use AI-based ERMs in the field of psychotherapy (17). However, first promising findings indicated high levels of accuracy for automated continuous emotion recognition and significant consistency with manual ratings in psychotherapy (17, 24).

To address the challenges of assessing emotions in naturalistic settings, the open-source software *Non-Verbal Behavior Analyzer* [NOVA; (29–32)] has been adapted to psychotherapy research because it does not interfere with the therapeutic process. NOVA is a software originally developed as an interview analysis tool within the EU FP-7 project TARDIS [2012–2015, (33)]. It was extended to include interactive machine learning capabilities as part of the ARIA-VALUSA Horizon project [2015–2018, (34)]. NOVA is an open-source tool and available on GitHub^1^. In a pilot study, it was applied to 72 therapy sessions of a test anxiety treatment (29). NOVA was evaluated as intuitive and ergonomic by trained human raters. Furthermore, they highlighted the wide range of functions and its good usability.

In summary, the progressive advancement of AI-based software solutions enables new assessment methods and fields of investigation. Continuous video-based emotion recognition can now be applied resource-efficiently and non-invasively in naturalistic psychotherapeutic settings. Therefore, this study aimed to apply NOVA to psychotherapy research and evaluate the validity of this method for assessing emotional expressions. For this purpose, the associations between the average arousal and valence of the patient per session determined by means of NOVA on the one hand, and the emotion assessment by the patient and therapist as well as symptom severity and the established process variables emotional, interpersonal and coping experiences (12), which are based on Grawe’s (35) process variables, on the other hand were to be examined. Therefore, we investigated the following hypotheses:

1. Emotion recognition models comparison: Valence and arousal estimated by the ERM correlate positively with human coders’ ratings. ERMs using different feature sets differ in terms of the strength of their correlation with human ratings of arousal and valence. The best ERM with the highest positive correlation can be identified.
2. The patients’ emotions (identified using the superior ERM in NOVA) are related to the patient and therapist ratings of the patients’ emotions at the end of the session using the PANAS. We expected higher negative valence to be correlated with more sadness, shame, anxiety, and anger. Accordingly, we expected higher positive valence to be associated with more satisfaction, energy, and relaxation. Furthermore, we expected higher arousal to be correlated with more anger, satisfaction, and energy. Lower arousal was expected to be correlated with more relaxation and sadness.
3. The patients’ emotions (identified using the superior ERM in NOVA) are related to the three process variables affective experience, interpersonal experience, and coping experience. We expected stronger affective experiences to be associated with higher arousal and more negative valence. Furthermore, we expected stronger interpersonal and coping experiences to be associated with more positive valence.
4. Emotion recognition models-rated arousal and valence are predictors of patient symptom severity in the respective session and the two following sessions (comprising a period of around 2 weeks). More arousal and more negative valence are associated with higher symptom severity.

## Materials and methods

### Participants and treatment

**FIGURE 1:**
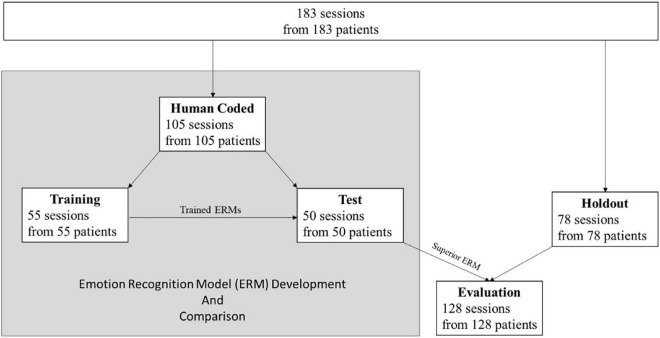
Flowchart of the sample distribution.

All patients in this study were treated with integrative cognitive-behavioral therapy (CBT) between 2017 and 2019 at an outpatient clinic in southwest Germany. The following inclusion criteria had to be met: (1) At least ten therapy sessions, and (2) patients older than 16 years. Exclusion criteria were: (1) Organic, including symptomatic mental disorders (ICD-10: F00-F09), (2) primary diagnosis of schizophrenia, schizotypal, or delusional disorders (ICD-10: F20-F29) because of expected problems during coding due to the peculiarities of the affective processing of these disorders (36), (3) physical limitations that interfere with the expression of emotions (e.g., paralysis, prostheses, amputations, dystonia, rigidity, burn, and disfigurement), and (4) patient transfer to a different therapist during the course of therapy. In total, videos of 183 patients (one session/video per patient) were used in this study. Videos were selected randomly from a sample with high video quality. One video per patient from a randomly varying session was included in this study. The training sample consisted of 55 videos, which were coded twice by different coders. The test sample consisted of further 50 patients who were not part of the training sample. The final application sample consisted of the 50 sessions from the test sample plus 78 additional patients (see Figure 1). Patients in the application sample had on average 35.68 (*SD* = 18.47) sessions of psychotherapy. They were between 18 and 72 years old with a mean of 34.72 years (*SD* = 12.77) and 70 (54.7%) of them were female. Detailed sample characteristics of the training, test, and evaluation samples can be found in Table 1. All therapists participated in a 3-year (full-time) or 5-year (part-time) postgraduate training program with a CBT focus and had received at least 1 year of training before beginning to see patients. They were supervised by a senior therapist every fourth session and were supported by a feedback system monitoring patient outcomes on a session-by-session basis (37). Therapists were trained in an integrative CBT approach including interpersonal and emotion-focused elements (35, 38, 39). All therapists were familiar with various disorder-specific CBT manuals but individually adapted their approach depending on patients’ characteristics. Psychometric feedback was provided to therapists after each session [for a detailed description of the treatment setting see (40)]. Research data were routinely collected via a range of instruments and all therapy sessions were videotaped. The patients were informed in writing when they registered for therapy and in person during the first therapy session about the continuous video recording as well as the collection of psychometric data. They were also informed about the evaluation of the video and psychometric data and the anonymized publication. Patients were informed about their right to withdraw their consent. All patients gave their written informed consent. This procedure was approved by the Ethics Committee of the German Psychological Society (DGPs, 2020-03-20VADM).

**TABLE 1:** Sample characteristics of the training, test, and evaluation sample.

	Training (*n* = 55)		Test (*n* = 50)		Evaluation (*n* = 128)	
	Mean (*SD*) or %	Range or *N*	Mean (*SD*) or %	Range or *N*	Mean (*SD*) or %	Range or *N*
Patient age (years)	32.88 (13.34)	18–69	34.64 (13.42)	18–65	34.72 (12.77)	18–72
Patient sex (female)	65.45	36	44	22	54.7	70
Therapy duration (sessions)	34.70 (36)	10–90	30.65 (13.86)	10–68	35.68 (18.47)	10–86
Drop-out rate	16.36	9	10	5	11.7	15
Comorbidity	52.73	29	44	22	39.8	51
**Primary diagnosis**						
Affective disorder	45.45	25	46	23	45.3	58
Anxiety disorder	10.91	6	8	4	10.3	13
Adjustment disorder	14.55	8	16	8	10.2	13
PTSD	9.09	5	8	4	7.8	10
Personality disorder	3.64	2	2	1	0.8	1
Other	16.36	9	20	10	25.6	33

Therapy duration refers to the complete length of the therapies from which the analyzed sessions originate.

Patients were diagnosed based on the Structured Clinical Interview for DSM-IV Axis I Disorders [SCID-I; (41)] conducted by intensively trained independent clinicians before actual therapy began. The interviews were videotaped and subsequently interviews and diagnoses were discussed in expert consensus teams comprised of four senior clinicians. Final diagnoses were determined by the consensual agreement of at least 75% of the team members. For the diagnosis of personality disorders, the International Diagnostic Checklist for Personality Disorders [IDCL-P; (42)] was conducted in the first sessions by the therapist.

### Automated emotion recognition using Non-Verbal Behavior Analyzer

#### Video processing

**FIGURE 2:**
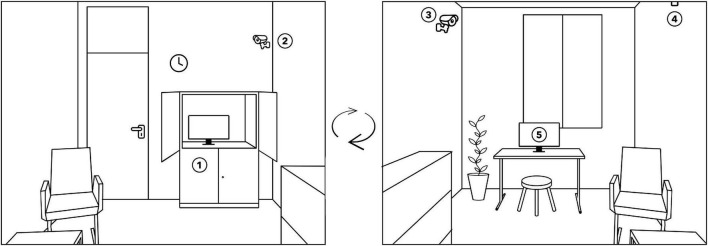
Sketch of the structure of a therapy room from two different angles. 1 = video and audio recording equipment (hidden in a cupboard), 2 = camera that focuses on the therapist, 3 = camera that focuses on the patient, 4 = microphone, and 5 = touchscreen for assessment of the session reports.

As a matter of routine, all therapy sessions were video recorded in the outpatient clinic. A sketch of the setting can be found in Figure 2. The therapist and patient were recorded separately by two different cameras. In this study, only the patient videos were considered. To ensure that the analyzed material covers the therapeutic interaction only, videos were checked, and additional video time was cut out. Afterward, video resolution was standardized to a scaled size of 640:480 and 25 frames per second and converted to mp4 format.

#### Software

**FIGURE 3:**
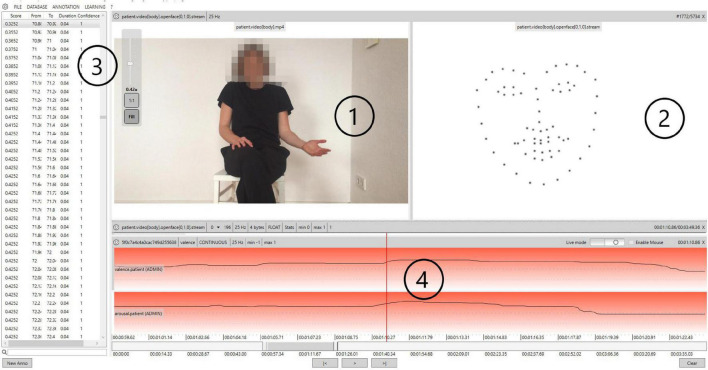
Exemplary representation of therapy videos in Non-Verbal Behavior Analyzer (NOVA). 1 = original video, 2 = schematic representation of the OpenFace feature points (not shown to raters during rating), 3 = time series for arousal/valence, and 4 = graphical representation of the arousal and valence characteristics/graphical interface for the rating of arousal/valence.

The central instrument for assessing arousal and valence was the NOVA software (29, 32). With the help of NOVA, arousal and valence were measured continuously between −1 and +1 with up to 16 decimal places. On the arousal dimension, −1 represents a drowsy state and +1 a state of strong agitation. On the valence dimension, −1 represents a very negative valence and +1 a very positive state. Both dimensions were then aggregated separately to generate mean values of arousal and valence per session. Without any additional training, NOVA is able to extract defined features of multiple modalities, such as the face and the body, so-called feature streams. In this study, different feature streams are used: OpenFace (43), a feature set that contains facial landmarks, head orientation as well as 17 actions units, OpenPose (44), a 2D skeleton tracking algorithm, as well as calculated features on the skeleton data such as the overall activation and the energy of the movements (31). An impression of the user interface of NOVA is given in Figure 3.

#### Rater training

To improve NOVA’s recognition performance, the algorithms were trained with manually rated videos (human coder ratings). The arousal and valence dimensions were scaled so that the values ranged from −1 to +1. All four raters (two female, two male) have graduated in psychology and underwent 30 h of training (consisting of technical instructions, information on common emotional theories with a special focus on the circumplex model, as well as video training material to train coding skills). Monthly supervision sessions were held with a licensed psychotherapist. Raters did not start rating in the project until their agreement in the example material was at least Cronbach’s α = 0.70.

### Psychometric questionnaires

#### Hopkins Symptom Checklist-11

The Hopkins Symptom Checklist-11 (HSCL-11) (41) is a short questionnaire based on the HSCL-25 (45), which is a short version of the Symptom Checklist-90 (46). It contains items from the subscales depression and anxiety. The HSCL-11 is a self-report questionnaire, which consists of 11 items, all structured as a 4-point Likert scale ranging from 1 (*not at all*) to 4 (*very*). In this study, the mean score for the whole questionnaire and scores for the subscales anxiety and depression were used. The internal consistency of the HSCL-11 (Cronbach’s alpha) was α = 0.85 (47). The mean of the 11 items is highly correlated with the Global Severity Index (GSI) of the Brief Symptom Inventory [BSI; *r* = 0.91; (12)]. Furthermore, psychometric properties are comparable to the BSI (45).

#### Subjective emotion rating

As a subjective emotion rating, an adaptation of the Positive and Negative Affect Schedule [PANAS, (48)] was carried out at the end of each session. The patient and therapist had to rate on a scale from 0 (*not at all*) to 100 (*extremely*) how sad, ashamed, frightened, angry, satisfied, energetic, and at ease the patient felt during the therapy session.

#### Session Report

At the end of each session, a short form of the Session Report (SR) (49–51) was administered. Patients assessed the subjectively perceived realization of process variables during the session. Three subscales, each ranging from −3 (*not at all*) to +3 (*yes, exactly*), were created from the 12-item version by averaging items of a scale: coping experiences (6 items), interpersonal experiences (4 items), and affective experiences (2 items; for more details see Rubel et al. (12)). The internal consistency (Cronbach’s alpha) was shown to be α = 0.89 for coping experiences, α = 0.90 for interpersonal experiences, and α = 0.85 for affective experiences (12).

### Data analytic strategy

*Hypothesis 1: ERM comparison – Identifying the best NOVA ERM*

The human and automated codings each resulted in continuous time series. In the first step, several ERMs were trained in the training data. Then, they were tested in the training as well as in the test data for their agreement with human ratings by correlating automatic (via NOVA) and human ratings for each session. Correlation coefficients were averaged over all sessions. Furthermore, the model fit indices mean squared error (MSE) and root mean square rrror (RMSE) were considered. The best performing ERM in training and test data was then applied to the application sample.

*Hypotheses 2–4: Evaluating the best NOVA ERM*

In the application sample, session scores for arousal and valence were calculated by taking a mean per session for each of the two emotion dimensions. Following hypothesis 2, Pearson correlations between the mean values of arousal and valence in NOVA and the subjective assessment of the patients’ emotions were carried out. Afterward, following hypothesis 3, mean session scores for arousal and valence were correlated with the patient assessment of the three process variables coping experiences, interpersonal experiences, and affective experiences using Pearson correlations. Furthermore, the predictive value of arousal and valence was tested using linear regression. Here, the dependent variable HSCL-11 (mean session scores as repeated measures of symptom severity) at session t, t+1, and t+2 was regressed on arousal and valence at session t as predictors (hypothesis 4).

## Results

### Human ratings’ reliability and test assumptions

The human coder ratings that were used to train the ERMs showed acceptable to excellent agreements on average for both arousal (Cronbach’s α = 0.73, ranging from α = 0.61 to α = 0.89) and valence (Cronbach’s α = 0.74, ranging from α = 0.60 to α = 0.90). Curves estimations indicated linearity of the data (all linear terms *p* < 0.05, all other terms *p* > 0.10). The Durbin Watson value of 1.774 for valence and 1.745 for arousal showed no evidence of autocorrelation of the predictor. The Shapiro–Wilk test showed that the residuals were normally distributed (valence: *p* = 0.302, arousal: *p* = 0.159). Homogeneity of variances was asserted using Levene’s test, which showed that equal variances could be assumed (all *p* > 0.10).

**Hypothesis 1: Valence and arousal estimated by the ERM correlate with human coders ratings. A best ERM can be identified.**

Different models based on different feature sets were evaluated. As expected, there were differences in the chosen models regarding their fit to the human codings. The model comparison showed that in both the training and test samples, the OpenFace model produced a higher agreement (training sample: *r*_*valence*_ = 0.26, *r*_*arousal*_ = 0.38; test sample: *r*_*valence*_ = 0.37, *r*_*arousal*_ = 0.44) to the human rating than the other models (see Table 2). Only in the test sample was a slightly higher agreement for valence (*r* = 0.42) for the OpenFace-Aureg ERM than for the OpenFace ERM (*r* = 0.37). However, the performance of the OpenFace ERM was close to the agreement for valence of the OpenFace-Aureg ERM and the OpenFace ERM outperformed all other models in assessing arousal. Therefore, this ERM was considered the best performing model.

**TABLE 2:** Emotion recognition model (ERM) comparison for the emotion dimensions arousal and valence based on different feature streams.

	Pearson CC	MSE	RMSE
**Valence**			
OpenFace	0.26	**< 0.01**	**0.07**
	**0.37**	**< 0.01**	**0.06**
OpenFace-aureg	0.26	<0.01	0.08
	0.42	<0.01	0.06
Openpose-activation	0.05	<0.01	0.09
	0.05	<0.01	0.07
Openpose-energy	<0.01	<0.01	0.09
	–0.03	<0.01	0.07
**Arousal**			
OpenFace	**0.38**	**< 0.01**	**0.10**
	**0.44**	**0.01**	**0.11**
OpenFace-aureg	0.34	<0.01	0.10
	0.10	0.02	0.13
Openpose-activation	0.05	0.01	0.11
	0.31	0.02	0.13
Openpose-energy	0.17	0.01	0.11
	0.20	0.01	0.12

The first line indicates the correlation between human rating and Non-Verbal Behavior Analyzer (NOVA) in the training sample. The second line indicates the correlation between human rating and NOVA in the test sample. Due to the large number of data points, *p*-values are not shown. Pearson CC, Pearson correlation coefficient; MSE, mean squared error; RMSE, root mean square error. Bold values indicate the best performing ERM.

**Hypothesis 2: The patients’ emotions (identified using the superior ERM in NOVA) are related to the patient and therapist ratings of the patients’ emotions at the end of the session.**

No significant correlations were found between the patient rating of his/her own emotions and the emotions recognized with the help of ERM. The therapist ratings of their patients’ emotions were significantly correlated with ERM-rated valence for sadness (*r* = −0.18, *p* < 0.049), shame (*r* = −0.23, *p* = 0.011), and anxiety (*r* = −0.21, *p* = 0.023), indicating that more positive ERM-coded emotions were associated with less sadness, less shame, and less anxiety of the patient rated by the therapist. Furthermore, ERM-rated arousal was significantly associated with the therapists’ assessment of their patients relaxation (*r* = −0.19, *p* = 0.039).

**Hypothesis 3: The patients’ emotions (identified using the superior ERM in NOVA) are related to the three process variables affective experience, interpersonal experience, and coping experience in the same and the two following sessions.**

In the third step, we examined the correlation between automatically recognized ERM-rated arousal and valence and the three processes of change interpersonal experiences, coping experiences, and emotional experiences. Significant associations could be found between valence and affective experience (*r* = −0.23, *p* = 0.010) and arousal and affective experience (*r* = 0.18, *p* = 0.044), indicating that more negative emotions and higher emotional arousal during the session were correlated with a higher level of emotional experience. Further results can be found in Table 3.

**TABLE 3:** Correlation between automatically recognized emotion dimensions and processes of change.

	Valence	Arousal	Interpersonal experience	Coping experience	Affective experience
Valence		0.24**	0.10	0.01	−0.23*
Arousal	0.24**		0.11	0.02	0.18*
Interpersonal experience	0.10	0.11		0.42**	0.21*
Coping experience	0.01	0.02	0.42**		0.13
Affective experience	−0.23*	0.18*	0.21*	0.13	

*N* = 128; *p ≤ 0.05, **p ≤ 0.01.

**Hypothesis 4: ERM-rated arousal and valence are predictors of patient symptom severity in the respective session and the two following sessions (comprising a time period of around 2 weeks).**

In the last step, the predictive value of ERM-recognized arousal and valence for symptom severity in the same (t) and the two following (t+1 and t+2) sessions was examined. There was no significant predictive effect for the HSCL-11 mean score measured at sessions t and t+2. There was a significant effect indicating that positive valence at session t predicted lower symptom severity in the following session (t+1; *b* = −5.38, β = −1.98, *SE* = 2.72, *t*_(126)_ = 1.98, *p* = 0.046). Valence and arousal did not predict symptom severity on the HSCL-11 subscale depression at any of the three time points. However, valence proofed as a stable predictor for the symptom severity on the HSCL-11 subscale anxiety over all three sessions (session t: *b* = −4.97, β = −0.20, *SE* = 2.39, *t*_(126)_ = −2.08, *p* = 0.038; t+1: *b* = −5.32, β = −0.18, *SE* = 2.88, *t*_(113)_ = −1.85, *p* = 0.066; t+2: *b* = −5.94, *SE* = 0.27, β = −0.21, *t*_(109)_ = −2.20, *p* = 0.026). Arousal did not predict the HSCL-11-score on the subscale anxiety to any of the three time points.

## Discussion

The automated recognition of emotions in psychotherapy enables novel insights into psychotherapy processes beyond self-report questionnaire data. AI-assisted emotion recognition allows for a cost-effective and time-saving emotion recognition over the course of entire naturalistic psychotherapy sessions. The present study demonstrated a potential application of the NOVA software and its contribution to expanding our understanding of mental processes and their correlates. In a first step (hypothesis 1), it could be shown that the ERM which was based on the whole OpenFace stream was best performing in the training and test sample except for valence in the test sample where the OpenFace-Aureg ERM performed slightly better. There was no noteworthy difference in performance of ERM between arousal and valence. All in all, the benefits of the OpenFace stream for emotion recognition, which have already been shown in several earlier studies (52–54) were confirmed.

Additionally, this study has shown the benefit of continuous recognition of arousal and valence in a naturalistic psychotherapy setting. Although there were no significant correlations between the patients’ ratings of their own emotions and the automated recognized dimensions of arousal and valence, arousal and valence correlated significantly with therapists’ ratings of sadness, shame, anxiety, and relaxation (hypothesis 2). This implies that the assessment of emotions using the NOVA software is most closely related to the external assessment of emotions, which is also consistent with the results of a previous study (55). This result was expected, as NOVA was trained with the help of external ratings and so reflects an external emotion recognition. Furthermore, it is known from previous studies that patients with mental disorders in particular find it difficult to adequately perceive, assess, and reflect emotions (4, 5, 7, 56). This might lead to limited results when it comes to self-report measurements. It is therefore questionable to what extent patient self-assessments can be used to validate the ERMs and we rather argue that what the AI-based ERMs assess goes beyond self-report data. Furthermore, it is noticeable that the emotions associated with the NOVA-rated valence all have negative connotations. One reason might be that the OpenFace ERM works well, especially with strong negative emotions. However, emotions such as joy, expressed rather reservedly in psychotherapy, are not yet adequately recognized. These findings should be a reason to further improve existing ERMs in NOVA. Another reason might be that human raters reacted more sensitive to strong negative emotions during the training process. Here, a further refinement of the training process for the recognition of positive valence could also contribute to the improvement of the ERMs.

Related to hypothesis 3, a significantly negative correlation between valence and the affective experience as well as a significantly positive association between arousal and the affective experience was found. These findings correspond to earlier results (13). They further support the interpretation that affective experience takes place when the patients’ problem is addressed in an affectively engaging way, resulting in high emotional involvement (12). At the same time, the ERM used in NOVA seems able to capture the affective experience as a combination of high arousal and negative valence which supports the validity of the used software and OpenFace ERM.

The predictive value of valence determined with NOVA for symptom severity could be shown for the anxiety subscale of the HSCL-11 over the respective and the two following sessions. This result shows that emotion recognition using NOVA has a predictive benefit for symptom severity that goes beyond the session itself. In particular, the anxiety scale of the HSCL-11 is associated with feeling strong emotions that are also expressed externally. It stands to reason that there is a correlation to this scale in particular. The depression scale, which is more associated with a reserved expression of emotions, shows no correlations with the emotion dimensions determined by NOVA. Therefore, NOVA could be used to assess and predict anxiety symptomatology based on the emotional expressions in patients’ faces only.

### Limitations

The results indicate that the used ERM should be improved. Therefore, it seems reasonable to increase the data sets used to develop and test the models. So far, it is not clear to what extent an overlap between human and machine ratings can be achieved since both might measure slightly different components of emotional expressions. The determination of the best ERM is based on descriptive differences between model fits, in particular due to the large number of data points. In future studies, the formulation of critical differences between correlation coefficients could be useful (57). It is possible that ERMs might capture emotions in a different way than human raters (hypothesis 1) or patients themselves (hypothesis 2).

Furthermore, it remains unclear whether it is more beneficial to train generalizable models in large heterogeneous multi-site samples or to train them on more selective homogeneous samples for each new population. While this study controlled for some diagnoses, some diagnostic heterogeneity was not avoidable. There could be certainly an effect of different diagnoses of interest that was not possible to examine in this feasibility sample. Furthermore, there might be differences in different psychotherapeutic settings. Therefore, in future studies, other settings like inpatient settings or online interventions could be an interesting field of application. This study has focused on between-patients statistics rather than within-patients statistics, which does not allow an assessment of individual changes or the computation of intra-individual effects. It was found that the level of group average compared to individuals can even lead to opposite relationships (e.g., relationship between self-efficacy and performance), showing that considering only group statistics can lead to wrong conclusions about the process that takes place within the individual (58–60). Hence, it might be helpful to investigate within-patients effects over the time of therapy to differentiate between time-varying effects and stable personality traits such as the individual expression of emotional states. The individual change in emotion during a therapy might be an important information correlated to outcome or therapeutic interventions. Finally, the present study does not consider interpersonal events between the two interaction partners. It is conceivable that, in addition to the intrapersonal emotional events of the patient, the interpersonal emotional fit between patient and therapist plays an important role in the therapeutic process and thus also for clinical improvements.

## Conclusion and future directions

Overall, this study can be seen as a step into a promising, innovative field of research in which methods of computer science can be used in naturalistic settings of psychotherapy. Further adaptations and validations of the underlying algorithms should take place. For this purpose, it may certainly be useful to combine data from different research institutions worldwide. For further validation, other constructs such as the recording of arousal via skin conductance could be used in future studies. Nowadays, physiological data can easily be collected with the help of a smartwatch, so that it is also possible to collect data in a natural therapy setting without any disturbing wiring (61). In addition to emotional facial expressions, para-verbal and verbal features from speech and text analysis (62, 63) as well as body movement data (64) might improve the accuracy and validity of emotion detection.

In the future, further validation studies should also be conducted to examine the different quality of EMRs in heterogeneous and homogeneous patient samples. It is possible that the correlation between ERMs coded emotional experience and treatment outcome varies as a function of the diagnostic group and initial symptom severity. From studies on therapist effects it is known that differences between therapists in their patients’ treatment outcome are dependent on initial impairment (65). Therapist effects have been shown to be larger for highly impaired patients compared to less impaired patients. Similarly, emotion expression might have a higher predictive value for more severely distressed patients, for whom the effectiveness of treatment may vary more than for less distressed patients.

Following the growing research in the field of interpersonal synchrony (64, 66–71), future research should go beyond the isolated consideration of patient emotions and instead compare time series of arousal and valence of the two dyadic partners, patient and therapist. Synchrony between patient and therapist, and its relation to therapeutic outcome and process variables has already been shown for movements (67, 72, 73), speech content and prosodic features (62, 74) as well as physiological measures (70, 75). Regarding emotions, it can also be assumed that it might be beneficial if patient and therapist are attuned to each other. At least, interpersonal emotional synchrony may provide important further insight into therapeutic processes.

## Data availability statement

The raw data supporting the conclusions of this article will be made available by the authors, without undue reservation.

## Ethics statement

The studies involving human participants were reviewed and approved by German Society for Psychology (DGPs). The patients/participants provided their written informed consent to participate in this study.

## Author contributions

PT, BS, TB, WL, and EA contributed to the conception and design of the study. PT, TB, DS, and EA organized the application of the NOVA software. PT and BS performed the statistical analyses. PT wrote the first draft of the manuscript. PT, TB, and BS wrote the sections of the manuscript. SE created illustrations. PT, BS, and SE were supported in the preparation of the revision by the co-authors. All authors participated in proofreading of the manuscript and read and approved the submitted version.

## Footnotes

1
https://github.com/hcmlab/nova
